# Increases and decreases in drug use attributed to housing status among street-involved youth in a Canadian setting

**DOI:** 10.1186/1477-7517-11-12

**Published:** 2014-04-10

**Authors:** Tessa Cheng, Evan Wood, Paul Nguyen, Thomas Kerr, Kora DeBeck

**Affiliations:** 1 Urban Health Research Initiative, British Columbia Centre for Excellence in HIV/AIDS, St. Paul's Hospital, 608-1081 Burrard Street, Vancouver, BC V6Z 1Y6, Canada; 2 Faculty of Health Sciences, Simon Fraser University, Blusson Hall, Room 11300, 8888 University Drive, Burnaby, BC V5A 1S6, Canada; 3 Faculty of Medicine, University of British Columbia, 317-2194 Health Sciences Mall, Vancouver, BC V6T 1Z3, Canada; 4 School of Public Policy, Simon Fraser University, 515 West Hastings Street, Suite 3271, Vancouver, BC V6B 5K3, Canada

**Keywords:** Homelessness, Drug use, Street-involved youth, Stable housing, Risk behaviour, Employment

## Abstract

**Background:**

Among a cohort of drug-using street-involved youth, we sought to identify the prevalence of reporting increases and decreases in illicit drug use due to their current housing status and to identify factors associated with reporting these changes.

**Findings:**

This longitudinal study was based on data collected between June 2008 and May 2012 from a prospective cohort of street-involved youth aged 14–26 in Vancouver, Canada. At semi-annual study follow-up visits, youth were asked if their drug use was affected by their housing status. Using generalized estimating equations, we identified factors associated with perceived increases and decreases in drug use attributed to housing status. Among our sample of 536 participants at baseline, 164 (31%) youth reported increasing their drug use due to their housing situation and 71 (13%) reported decreasing their drug use. In multivariate analysis, factors that were positively associated with perceived increases in drug use attributed to housing status included the following: being homeless, engaging in sex work and drug dealing. Regular employment was negatively associated with increasing drug use due to housing status. Among those who reported decreasing their drug use, only homelessness was significant in bivariate analysis.

**Conclusion:**

Perceived changes in drug use due to housing status were relatively common in this setting and were associated with being homeless and, among those who increased their drug use, engaging in risky income generation activities. These findings suggest that structural factors, particularly housing and economic opportunities, may be crucial interventions for reducing or limiting drug use among street-involved youth.

## Findings

### Introduction

Housing instability among street-involved youth remains a community and public health concern. Previous research has found that housing instability often precedes substance use [1] and is also linked to increased intensity of drug use [2], including initiation into injection drug use [3,4]. Furthermore, a loss of housing stability has been associated with higher intensity alcohol and crystal methamphetamine use among youth [5]. In contrast, residential stability appears to be protective against a range of risky drug and sexual behaviours [6,7].

Although housing is recognized to have an influence on substance use, few studies have explored whether youth attribute their housing status directly to changes in their drug use and if reporting this relationship is associated with risky behaviours or other factors. This study sought to identify the prevalence of street-involved youth who attribute changes in drug use to their housing status and factors associated with these relationships.

### Methods

Data for this study were obtained from the At-Risk Youth Study (ARYS), a prospective cohort study of street-involved youth in Vancouver, Canada. The study has been previously described in detail [8]. In brief, snowball sampling and extensive street-based outreach methods were employed. To be eligible, participants at recruitment had to be aged 14–26 years, use illicit drugs other than marijuana in the past 30 days, provide written informed consent and be street-involved, defined as being homeless (having no fixed address, sleeping on the street or staying in a shelter or hostel) or having used services designated for street-involved youth in the last 6 months [8-10]. At enrollment, and on a bi-annual basis, participants completed an interviewer-administered questionnaire that included questions related to demographic information and drug use patterns. At each study visit, participants were provided with a stipend (Canadian $20) for their time. The study has been approved by the University of British Columbia's Research Ethics Board.

For the present analyses, ARYS participants were eligible if they completed at least one study visit between June 2008 and May 2012. Our two outcomes of interest were ‘perceived increases in drug use attributed to housing status’ and ‘perceived decreases in drug use attributed to housing status’, where the term ‘housing status’ refers to a participant's housing situation (e.g. living in an apartment, a hotel, house, shelter or the street). Data for our outcomes were based on responses to the question: ‘Considering your drug use and your housing in the past 6 months, do you think that your housing situation affected your drug use?’ Participants who responded ‘yes’ or ‘sometimes’ were asked to specify if they thought they had generally ‘used more’ or ‘used less’ drugs in response to their housing situation. Youth who reported perceived increases in drug use were included in the outcome category for analysis 1, and those who perceived decreases in their drug use were included in the outcome category for analysis 2. Participants who perceived no change in their drug use were included as controls for both analyses. Given that our outcomes were determined by repeated measures, participants who changed their response over study follow-up may be included in both analyses.

Drug use patterns among the study sample were assessed using baseline data for the following: daily non-injection crystal methamphetamine use (yes vs. no), daily crack cocaine smoking (yes vs. no), daily non-injection cocaine use (yes vs. no) and daily non-injection heroin use (yes vs. no). In order to assess high-intensity drug use, we included binge drug use, defined as a period of using injection or non-injection drugs more often than usual (yes vs. no), and any injection drug use (yes vs. no). All drug use variables refer to activities in the past 6 months.

To identify factors associated with attributing housing status with our two outcomes of interest (perceived increases in drug use and decreases in drug use), we considered a number of explanatory variables of interest including the following socio-demographic factors: gender (female vs. male); age (per year older); ethnicity (Caucasian vs. other); currently being in a stable relationship, defined as being legally married, common law or having a regular partner (yes vs. no); and homelessness, defined as having no fixed address, sleeping on the street or staying in a shelter or hostel (as compared to ‘not homeless’, which included living in a house, apartment, single-room occupancy unit, treatment, recovery house or jail) (homeless vs. not homeless). Other variables considered included the following: regular employment, defined as having at least one source of income from a regular job, temporary work or self-employment (yes vs. no); engaging in sex work, defined as exchanging sex for money, drugs, gifts, food, clothes, shelter or favours (yes vs. no); and participation in drug dealing (yes vs. no). All behavioural variables refer to activities in the past 6 months.

Generalized estimating equations (GEE) with a logit link function and exchangeable correlation structure were used in two separate analyses to model factors associated with attributing housing status to perceived increases in drug use and decreases in drug use. The GEE method is a conventional analysis for longitudinal correlated within-subject data [11,12]. We performed bivariate GEE analyses to determine factors associated with perceived increases in drug use and decreases in drug use attributed to housing status. To adjust for potential confounding factors and identify factors that were independently associated with our two outcomes of interest (perceived increase in drug use and decrease in drug use), all variables that were significant at *p* < 0.10 in bivariate analyses were considered for inclusion in full multivariate models. For each multivariate model, a backward model selection procedure was used to identify the model with the best overall fit as indicated by the lowest quasilikelihood under the independence model criterion value [13]. All statistical analyses were performed using the SAS software version 9.3 (SAS, Cary, NC, USA). All *p* values are two-sided.

### Results

During the study period, 561 participants were recruited into ARYS, among whom 536 answered all questions relevant for this analysis. This group included 171 (32%) women and 352 (66%) persons of Caucasian ethnicity, and the median age of participants was 22 years (interquartile range [IQR] = 20–24). Among our sample at baseline, 164 (31%) attributed increased drug use to their housing status, 301 (56%) reported no change and 71 (13%) reported a decrease in drug use due to their housing status. Over the study period, 261 (49%) participants perceived an increase in their drug use due to their housing situation, 425 (79%) perceived no change in their drug use and 198 (37%) perceived a decrease in their drug use. The overall number of study observations collected over the study period was 1,724, with 387 (22%) including a report of increased drug use due to housing status, 1,056 (61%) including a report of no change and 281 (16%) including a report of decreased drug use due to housing status. Among the analytic sample of 536, 382 (71%) participants had at least one follow-up visit, with a median number of follow-up visits of 3 (IQR = 2–4) and median duration of time under study follow-up of 28 months (IQR = 17–35). Analyses of whether the participants who contributed more than one study visit were significantly different with regard to age, gender or homelessness from those with just one study visit revealed no statistically significant differences between the two groups (all *p >* 0.10).

The characteristics and drug use patterns of this study sample at their first study visit during the study period are presented in Table 1, stratified by the attributed effect of drug use on housing status. The bivariate and multivariate GEE analyses of socio-demographic, behavioural and other risk variables associated with perceived increases in drug use are presented in Table 2. In multivariate analysis, factors that remained positively associated with perceived increases in drug use included the following: homelessness (adjusted odds ratio [AOR] = 2.45, 95% confidence interval [CI] 1.89–3.17), involvement in sex work (AOR = 1.65, 95% CI 1.02–2.67) and drug dealing (AOR = 1.79, 95% CI 1.38–2.31), while regular employment was negatively associated with increased drug use (AOR = 0.77, 95% CI 0.60–1.00). In the bivariate analyses for those who perceived decreases in their drug use (Table 3), homelessness was the only significant factor (odds ratio = 1.60, 95% CI 1.20–2.11), and therefore, a multivariate model was not constructed for this outcome.

**Table 1:** **Characteristics of street-involved youth at baseline (*n*** **= 536)**

		**Drug use affected by housing**^**a**^		
	**Total (%)**	**Drug use increased (%)**	**No change in drug use (%)**	**Drug use decreased (%)**
	**(*n*** **= 536)**	**(*n*** **= 164)**	**(*n*** **= 301)**	**(*n*** **= 71)**
Characteristic				
Age				
Median (IQR)	22 (20–24)	22 (20–24)	21 (19–23)	22 (20–24)
Gender				
(Female vs. male)	171 (31.90)	53 (32.32)	100 (33.22)	18 (25.35)
Caucasian ethnicity				
(Yes vs. no)	352 (65.67)	110 (67.07)	194 (64.45)	48 (67.61)
Stable relationship (currently)				
(Yes vs. no)	190 (35.45)	62 (37.80)	103 (34.22)	25 (35.21)
Homeless^a^				
(Homeless vs. not homeless)	357 (66.60)	131 (79.88)	177 (58.80)	49 (69.01)
Employment^a^				
(Yes vs. no)	228 (42.54)	58 (35.37)	134 (44.52)	36 (50.70)
Sex work^a^				
(Yes vs. no)	41 (7.65)	16 (9.76)	21 (6.98)	4 (5.63)
Drug dealing^a^				
(Yes vs. no)	244 (45.52)	84 (51.22)	125 (41.53)	35 (49.30)
Drug use variables				
Daily crystal meth use^a,b^				
(Yes vs. no)	45 (8.40)	22 (13.41)	19 (6.31)	4 (5.63)
Daily crack smoking^a,b^				
(Yes vs. no)	86 (16.04)	37 (22.56)	39 (12.96)	10 (14.08)
Daily cocaine use^a,b^				
(Yes vs. no)	12 (2.24)	6 (3.66)	5 (1.66)	1 (1.41)
Daily heroin use^a,b^				
(Yes vs. no)	20 (3.73)	7 (4.27)	11 (3.65)	2 (2.82)
Binge drug use^a,c^				
(Yes vs. no)	180 (33.58)	69 (42.07)	91 (30.23)	20 (28.17)
Injection drug use^a^				
(Yes vs. no)	167 (31.16)	56 (34.15)	89 (29.57)	22 (30.99)

^a^Refers to activities in the past 6 months; ^b^refers to non-injection drug use; ^c^refers to injection and non-injection drug use.

**Table 2:** **Bivariate and multivariate GEE analyses of factors associated with perceived increases in drug use (*n*** **= 511)**

**Characteristic**	**Odds ratio (95% CI)**	***p *** **value**	**Adjusted odds ratio (95% CI)**	***p *** **value**
Age				
(Per year older)	0.99 (0.95–1.04)	0.788		
Gender				
(Female vs. male)	0.90 (0.67–1.22)	0.503		
Caucasian ethnicity				
(Yes vs. no)	1.32 (0.99–1.77)	0.056	1.34 (0.99–1.82)	0.055
Stable relationship (currently)				
(Yes vs. no)	0.98 (0.78–1.24)	0.882		
Homeless^a^				
(Homeless vs. not homeless)	2.64 (2.05–3.39)	<0.001	2.45 (1.89–3.17)	<0.001
Employment^a^				
(Yes vs. no)	0.78 (0.61–1.00)	0.051	0.77 (0.60–1.00)	0.047
Sex work^a^				
(Yes vs. no)	1.64 (1.01–2.67)	0.045	1.65 (1.02–2.67)	0.041
Drug dealing^a^				
(Yes vs. no)	1.98 (1.54–2.54)	<0.001	1.79 (1.38–2.31)	<0.001

^a^Refers to activities in the past 6 months.

**Table 3:** **Bivariate GEE analyses of factors associated with perceived decreases in drug use (*n*** **= 468)**

**Characteristic**	**Odds ratio (95% CI)**	***p *** **value**
Age		
(Per year older)	1.03 (0.98–1.08)	0.209
Gender		
(Female vs. male)	0.93 (0.68–1.28)	0.657
Caucasian ethnicity		
(Yes vs. no)	1.27 (0.93–1.74)	0.140
Stable relationship (currently)		
(Yes vs. no)	1.20 (0.90–1.59)	0.213
Homeless^a^		
(Homeless vs. not homeless)	1.60 (1.20–2.11)	0.001
Employment^a^		
(Yes vs. no)	1.08 (0.81–1.43)	0.618
Sex work^a^		
(Yes vs. no)	1.09 (0.61–1.95)	0.776
Drug dealing^a^		
(Yes vs. no)	1.20 (0.89–1.63)	0.228

^a^Refers to activities in the past 6 months.

### Discussion

In the present study, 22% of study observations included a report of perceived increase in drug use, 16% included a report of perceived decrease in drug use and 61% reported no change in drug use due to the participant's current housing situation. Homelessness and prohibited income-generating activities, specifically drug dealing and sex work, were positively associated with perceived increases in drug use, while regular employment was negatively associated with perceived increases in drug use attributed to housing status. Homelessness was the only factor associated with perceived decreasing drug use attributed to housing status in the bivariate analysis.

The relationship between homelessness and perceived increases in drug use is consistent with existing research suggesting that homeless youth are more likely to engage in riskier and more frequent substance use than housed youth [5,14]. Specifically, prior findings indicate that some homeless youth may increase their drug use as a survival strategy to suppress their appetite when food is limited and to stay alert to protect themselves and their belongings [15]. Problematic drug use and homelessness have been identified as accelerating entrenchment in illicit drug scenes [16], which is especially concerning since high-intensity drug use was prevalent in our sample and homeless youth have been found to be more likely to have difficulty accessing drug and alcohol treatment services [17].

Homelessness was also positively associated with perceived decreases in drug use in the bivariate analyses. One potential interpretation of this association is that homeless youth have less stability and face greater challenges generating income, which may make it harder to purchase drugs and could result in reduced drug consumption. Given that only homelessness was associated with reported decreasing drug use and a multivariate analysis could not be constructed to address potential confounding factors, further investigation is needed to better contextualize the relationship between homelessness and reduced drug use.

The relationship between perceived increased drug use to housing status and high-risk behaviours, such as sex work and drug dealing, suggests that these youth are more likely to be economically vulnerable. Contributing factors to economic vulnerability include substance use and lower levels of education, which are themselves associated with engaging in the drug and sex trade [18-22]. As active involvement in the street economy is linked to substance abuse, disengagement with the labour market is similarly associated with a range of psychological and health-related harms such as social exclusion and estrangement [23,24] and increased substance use [25]. Given that our findings and the research suggest that employment may play a protective role against increasing substance use, providing economic security to street-involved youth may mitigate some of the harmful health-related effects of homelessness.

There are several limitations to this study. First, these data are observational, and therefore, we are cautious about drawing causal inferences. Second, even though extensive street-based outreach was used, ARYS participants were not systematically recruited, and therefore, the generalizability of this study may be limited. The demographics of the sample, however, are consistent with other samples of street-involved youth in Vancouver [26,27]. Third, our measure for the main outcomes of interest is the perception of participants. Consequently, this may underestimate or overestimate the effect of housing on drug use depending on whether youth are unaware of the impact of housing on their substance use or if they over-attribute the role of housing to their drug use. Finally, this study focused on the reported influence of housing status on drug use and did not examine the potential effect of drug use on housing status. Drug use has been identified as a determinant of housing [3,4], and there is a need to better understand the complex relationships affecting street-involved youth's housing and drug use.

In summary, our study indicates that street-involved youth perceive their drug use to be affected by their housing status, and a range of social and structural factors are associated with this relationship, including economic vulnerability. These results suggest that efforts should be made to reduce the social and structural barriers to meaningful employment for street-involved youth. Policy approaches focusing on appropriate housing and reducing economic vulnerability show potential for addressing the trajectories of drug use among homeless and street-involved youth.

## Competing interests

The authors declare that they have no competing interests.

## Authors’ contributions

TC took on a primary role in conceptualizing and designing the study, conducting the analysis and preparing the manuscript. PN assisted in conceptualizing the statistical analyses and performed all statistical analyses. PN also reviewed the final manuscript for statistical accuracy. EW and TK assisted with the interpretation of the data and revised the manuscript for important intellectual content. KD took on a senior role in conceptualizing and designing the study and reviewing drafts of the manuscript. All authors reviewed and approved the final manuscript.
